# TAT-mediated transduction of bacterial redox proteins generates a cytoprotective effect on neuronal cells

**DOI:** 10.1371/journal.pone.0184617

**Published:** 2017-09-08

**Authors:** Cecilia L. Balaban, Claudia Banchio, Eduardo A. Ceccarelli

**Affiliations:** Instituto de Biología Molecular y Celular de Rosario (IBR), CONICET, Facultad de Ciencias Bioquímicas y Farmacéuticas, Universidad Nacional de Rosario, Ocampo y Esmeralda, Rosario, Argentina; University of Nebraska-Lincoln, UNITED STATES

## Abstract

Cell penetrating peptides, also known as protein transduction domains, have the capacity to ubiquitously cross cellular membranes carrying many different cargos with negligible cytotoxicity. As a result, they have emerged as a powerful tool for macromolecular delivery-based therapies. In this study, catalytically active bacterial Ferredoxin-NADP^+^ reductase (LepFNR) and Heme oxygenase (LepHO) fused to the HIV TAT-derived protein transduction peptide (TAT) were efficiently transduced to neuroblastoma SHSY-5Y cells. Proteins entered the cells through an endocytic pathway showing a time/concentration dependent mechanism that was clearly modulated by the nature of the cargo protein. Since ferredoxin-NADP^+^ reductases and heme oxygenases have been implicated in mechanisms of oxidative stress defense, neuroblastoma cells simultaneously transduced with TAT-LepFNR and TAT-LepHO were challenged by H_2_O_2_ incubations to judge the cytoprotective power of these bacterial enzymes. Accumulation of reactive oxygen species was significantly reduced in these transduced neuronal cells. Moreover, measurements of metabolic viability, membrane integrity, and cell survival indicated that these cells showed a better tolerance to oxidative stress. Our results open the possibility for the application of transducible active redox proteins to overcome the damage elicited by oxidative stress in cells and tissues.

## Introduction

Plasma and organelle membranes of eukaryotic cells constitute rigorous barriers that selectively control the movement of substances. Most of the exogenous compounds that could endanger the essential cell homeostasis are impermeable under physiological conditions. Nevertheless, these fences hamper the vast majority of hydrophilic drugs to reach their target molecules inside the cell. Many transcription factors, enzymes, peptides, small interfering RNAs (siRNAs) and oligonucleotides have become very attractive targets for overcoming different diseases and malignancies. Nevertheless, they all require delivery strategies to circumvent the membrane obstacle [1]. There are a variety of delivery techniques that include microinjection, electroporation or liposome transfection and the use of viral based vectors. However, more robust and safer uptake alternatives are unfortunately still lacking.

Promising approaches to delivering macromolecules into cells emerged almost 30 years ago from two unexpected findings: the HIV TAT transactivating factor [2,3] and the Drosophila Antennapedia transcription factor [4] were shown to translocate cell membranes and enter cells. The intriguing spontaneous uptake of both proteins led to structure/function studies to find the minimal amino acid sequence required to support protein import. Therefore, it was determined that TAT-PTD (TAT-Protein Transduction Domain), a short positively charged, arginine-rich amino acid peptide, was the main contributor to HIV TAT protein transduction [5]. Since then, these non-invasive vectors known as “cell penetrating peptides (CPPs)” or “PTDs” have promoted numerous advances in macromolecular delivery-based therapies.

In recent years, several studies have given an indication of the wide delivery power of TAT-PTD by showing the uptake of many different cargos (such as proteins, oligonucleotides, nanoparticles and drugs) with low cytotoxicity in cultured cells and animal models [6–8]. Protein transduction often involves a three-step process: first, binding of the PTD to the cellular membrane; second, stimulation of cellular uptake by endocytosis; and third, endosomal escape of cargo into the cytoplasm [6,9]. Alternative internalization pathways, such as direct penetration of the membrane, have been proposed as well [10]. Evidence indicates that the route of entry of arginine-rich peptides may differ according to the experimental conditions (e.g. peptide concentration [11], cargo characteristics [12,13], etc.). Consequently, the precise membrane translocation mechanism is still a matter of controversial debate.

Reactive oxygen species (ROS) management became central for every living organism on earth. Oxidative metabolism requires a very fine tuning to guarantee cell homeostasis, limiting ROS formation and regulating antioxidant defenses. Oxidative stress implies that this balance has been altered or lost, leading to the overproduction of ROS and followed by internal cell structure damage.

Neurons are particularly vulnerable to oxidative stress since they exhibit higher oxygen consumption compared to other tissues and contain highly peroxidizable substrates. Moreover, they have a long-life span during which some of them, located in specific regions related to motor activity, accumulate iron, a main contributor to ROS generation [14,15]. Furthermore, the brain has lower antioxidant defenses when compared to other tissues, for example, nearly 10% of antioxidants found in the liver [16,17]. Therefore, exogenous antioxidant therapies are considered a valuable approach in the prevention of neurodegeneration. Recently, research conducted in human brain cortical neurons has revealed that TAT mediated transduction of Peroxiredoxin 6 conferred resistance against oxidative stress inducers [18].

Ferredoxin-NADP(H) reductases (FNR, EC 1.18.1.2) integrate a family of monomeric enzymes that contain noncovalently bound FAD as a prosthetic group. These flavoproteins catalyze the reversible electron transfer between NADP(H) and electron carrier proteins like ferredoxin or flavodoxin. FNR isoforms were found in every living organism that was examined in their quest, participating in a broad spectrum of redox reactions [19]. In addition to their involvement in photosynthesis and other pathways of central metabolism, FNRs have been previously investigated as likely tools to mitigate the effect of oxidative stress [20–22]. Heme oxygenases (HO) are evolutionarily conserved enzymes that catabolize heme into equimolar amounts of labile Fe, carbon monoxide, and biliverdin. Heme, the substrate of HO, exists essentially as a prosthetic group of hemoproteins as a resource that allows the incorporation of Fe^2+^ into proteins. Free heme is a dangerous agent inside the cell as it can catalyze the production of aggressive ROS through the Fenton reaction [23]. Under homeostasis this effect is strictly controlled by the binding of heme into the heme pockets of hemoproteins. However, under oxidative stress, some hemoproteins can release their heme prosthetic groups, producing free heme. HOs are thought to protect against various types of oxidative stress, as previously described in detail [24]. Additionally, the products of HO activity are biologically active molecules that possess diverse antioxidant effects.

In pathogenic microorganisms, HOs are involved in the iron acquisition from the host heme during infection [25] and on the protection of the pathogen against heme toxicity [26]. Previous work from our group has demonstrated that HO from *Leptospira interrogans* (LepHO), a parasitic bacterium that infects humans and causes leptospirosis, is able to bind and efficiently catalyze the cleavage of heme to free iron and biliverdin. More interestingly, LepHO uses a bacterium Ferredoxin NADP^+^ reductase (LepFNR) and NADPH as the sole electron source. This enzyme pair is highly stable, quite unique and extremely efficient, and will degrade heme to biliverdin in solution with the only requirement of NADPH, a substrate readily available in the cellular cytoplasm and in the organelles [27]. Consequently, we hypothesized that the delivery of this redox pair into cells during an oxidative stress challenge may provide the defense reinforcement that, for particularly vulnerable cells like neurons, could mean the chances of survival.

In this study, TAT fusions to LepFNR and LepHO expressed in *E*. *coli* proved to be capable of degrading heme to biliverdin *in vitro*. Both fusion proteins efficiently transduced human neuroblastoma cells (SHSY-5Y) in culture in a time/concentration-related fashion. Moreover, transduction mediated by TAT-PTD was clearly modulated by the cargo protein and showed indication of an endocytic entry pathway. This redox pair could simultaneously translocate neuronal cells and generate a cytoprotective effect in response to oxidative stress *in vitro*.

## Materials and methods

### Construction of expression vectors

The genes encoding LepHO and LepFNR from *L*. *interrogans* serovar Lai 56601 were amplified from a pET-TEV vector harboring the LepHO sequence [27] and the plasmid pET32JO-LepFNR [28] using the oligonucleotides depicted in the S1 Table. Oligonucleotide sequences were designed to introduce BamHI and HindIII (LepHO) and BamHI and SacI (LepFNR) restriction enzymes sites at the 5’ and 3’ ends, respectively. The expression plasmids were constructed by inserting the amplification products previously cut with the indicated enzymes, into the similarly restricted pET-TEV plasmid. To obtain the TAT fused proteins, two oligonucleotides were synthesized and annealed to generate a double-stranded oligonucleotide encoding 11 amino acids from the basic domain of HIV-1 TAT (YGRKKRRQRRR) and NdeI and BamHI restriction sites at 5’ and 3’ ends respectively (S1 Table). The double-stranded oligonucleotides were ligated into NdeI-BamHI–digested pET-TEV vector in frame with a 6 His open-reading frame to generate pHisTAT-LepHO and pHisTAT-LepFNR.

### Protein expression and purification

His-Tag LepHO/TAT-LepHO and LepFNR/TAT-LepFNR were expressed and purified essentially as reported previously [27,28]. The purified protein was stored at −80°C until used.

### TAT-LepFNR/TAT-LepHO electron transfer reaction

Heme turnover was spectrophotometrically determined as described before. Measurements were carried out in 600 μl of 25 mM HEPES-KOH (pH 7.5) at 25°C in a cell with 1 cm path length. Reaction mixture contained 6 μM TAT-LepHO, 0.1 mg/mL catalase (Sigma), 3 mM glucose 6-phosphate, 300 μM NADP^+^, 1 unit/mL glucose-6-phosphate dehydrogenase and 0.5 μM TAT-LepFNR. Optical absorption spectra were obtained using a Shimadzu UV-2450 spectrophotometer. Spectral changes between 300 and 800 nm were monitored over a 30 min time period. Biliverdin formation was followed using the absorbance change at 680 nm [27].

### Transduction of fusion proteins into SH-SY5Y human neuroblastoma cells

SH-SY5Y human neuroblastoma cells (ATCC CRL-2266) were maintained in Dulbecco's Modified Eagle's Medium/Nutrient F-12 Ham (DMEM-F12) (Gibco) containing 10% fetal bovine serum (FBS), and antibiotics (100 mg/mL streptomycin, 100 U/mL penicillin) at 37°C in a humidified atmosphere of 95% air and 5% CO_2_. To study the transduction of TAT fused proteins, 7.5 x 10^5^ cells were seeded in 35 mm plates and cultured for 24 h. Monolayers of cells were incubated for selected time intervals with TAT-fused proteins in serum-free DMEM-F12. After treatment, cells were washed twice with PBS buffer and treated with trypsin (0.2% trypsin, 0.25 mM EDTA) for 15 min to degrade unincorporated protein. Finally, cells were harvested by centrifugation (1000xg, 5 min) and pellets were resuspended in RIPA lysis buffer (50 mM Tris-HCl (pH 8.0), 150 mM NaCl, 0.5% Tritón X-100, 0.5% Sodium deoxycholate, 0.1% SDS) with protease inhibitors benzamidine (1 mM) and PMSF (1 mM).

### Western blot analysis

Cell extracts were prepared by two rounds of sonication with a biorruptor^TM^ (Diagenode, UCD-200) followed by centrifugation at 12000xg for 20 min. Supernatant total protein concentration was determined using a Pierce BCA® protein assay kit (Thermo Scientific). 50–60 µg of total protein were separated by 12% SDS-PAGE and transferred onto a nitrocellulose membrane (Amersham) for Western blotting [29], using primary antibodies against LepHO (1:750), LepFNR (1:100) raised in rabbits immunized with recombinant LepHO o LepFNR. Antibodies against βIII-Tubulin (1:6000) were from Sigma, St. Louis, MO, USA. Subsequently, blots were developed with a goat anti-rabbit phosphatase-conjugated IgG (Sigma, St. Louis, MO, USA) and nitro-blue tetrazolium and 5-bromo-4-chloro-3'-indolyphosphate as chromogenic substrates.

### Immunofluorescence and confocal microscopy

Cells were grown on glass coverslips in 35 mm plates for 24 h. After incubation with TAT-fused proteins (3 μM) in serum-free DMEM-F12, cells were thoroughly washed 4 times with PBS and fixed with 4% PFA-Sucrose in PBS for 30 min. Fixed cells were permeabilized with 0.2% Triton X-100 in PBS and subsequently blocked with 0.5% BSA in PBS for 1 h. Cells were then incubated with rabbit anti-LepFNR (1:50) and anti-LepHO (1:200) antibodies for 1 h at room temperature. The coverslips were washed, incubated 1 h at room temperature with the secondary Alexa-488 conjugated anti-rabbit antibody (1:800, Life Technologies Corporation, Carlsbad, CA, USA) and mounted with ProLong® with DAPI. A sample was processed in parallel omitting the primary antibody as a background control. Confocal images were obtained using a Zeiss LSM 880 confocal microscope with a 20X objective. Zen image acquisition software (Carl Zeiss) was used to analyze images.

### Metabolic viability assay

SH-SY5Y cells were seeded in 96-wells plates at a density of 3x10^4^ cells/well in 100 μL serum-free medium. Cells were treated with H_2_O_2_ (100 μM) for 6 h. Then, without removing the oxidizing agent, TAT-LepHO and TAT-LepFNR (3 μM) were simultaneously added to the medium and the cells incubated for a further 18 h, reaching a total incubation time of 24 h. Control cells were maintained in identical conditions without the addition of the fusion proteins. At the end of the incubation time, culture media was replaced with 100 μL of 3-(4,5-dimethylthiazol-2-yl)-2,5-diphenyl tetrazolium bromide (MTT) and after 4 h the formazan crystal produced was dissolved in DMSO and absorption at 540 nm was determined. Cell viability was expressed as a percentage of control cells MTT reduction. Three wells per group were assayed in each experiment.

### Membrane integrity assay

Cell membrane integrity was evaluated through Lactate Dehydrogenase leakage into the culture medium using LDH activity reagent (0.6 mM Sodium pyruvate, 0.18 mM NADH, 50 mM Sodium Phosphate Buffer (pH 7.5)). SH-SY5Y cells were seeded in 96-well plates at a density of 3x10^4^ cells/well in 100 μL serum-free medium. Cells were treated with H_2_O_2_ (100 μM) for 6 h. Then, without removing the oxidizing agent, TAT-LepHO and TAT-LepFNR (3 μM) were simultaneously added to the medium and the cells incubated for a further 18 h, reaching a total incubation time of 24 h. Control cells were maintained in identical conditions without the addition of the fusion proteins. To calculate LDH activity, aliquots of media and warm reagent were mixed in a 96-wells plate, and NADH absorbance decay was recorded at 340 nm during 4 min at 30 s intervals using a microplate spectrophotometer system (Epoch, BioTek). Data were express as a percentage of LDH release with respect to total LDH activity. Medium of cells treated with RIPA buffer represented total LDH activity. Three wells per group were assayed in each experiment.

### Vital staining

SH-SY5Y cells in suspension (2x10^6^) were simultaneously treated with TAT-LepHO and TAT-LepFNR (3 μM) or left in serum-free medium for 1 h and then exposed to H_2_O_2_ (1 mM) for another hour. Cell pellets were washed with PBS buffer and resuspended in 500 μL of staining medium (serum free DMEM-F12, 2 μg/mL Fluorescein Diacetate, 1.2 μg/mL Propidium Iodide) during 5 min in a dark place at room temperature. Cells were washed with 1 mL PBS buffer and resuspended in 200 μL PBS buffer. The cell suspension (10 μL) was placed on a microscope coverslip and observed under a fluorescence microscope (Eclipse E800 microscope, Nikon). Quantification was made using cell counting software (ImageJ) on 3 image fields of each independent experiment. Images for quantification were taken with a 20X objective.

### Measurement of intracellular ROS

SH SY5Y cells (7.5x10^5^) were seeded in 35 mm culture plates and incubated 24 h in complete medium. Cell monolayers were pre-treated for 2 h with H_2_O_2_ (500 μM). Then, the culture medium was discarded, and cells were loaded with 2’,7’-dichlorofluorescein diacetate (DCFH-DA, Sigma, St. Louis, MO, USA) (80 μM) for 30 min and subsequently washed with PBS buffer. Cells were either incubated in serum-free medium alone or treated with TAT-fused proteins (3 μM) plus Chloroquine (100 μM) for 1 h. After trypsinization, cells were resuspended in PBS buffer and transferred to a black 96 wells plate to read fluorescence at 520 nm (excitation: 485 nm) in a microplate reader Synergy (Biotek). Three wells per group were assayed in each experiment.

### Statistics

Data were analyzed with GraphPad Prism 5.0 software package and expressed as mean ± SD, experiments were performed in triplicate. These replicates were completely independent as they were done in different days, starting from different cell culture dishes. Statistical analyses were performed using one-way ANOVA followed by the Tukey’s post hoc test for comparison between means. Statistical significance was set at p < 0,05.

## Results

### Expression and purification of the LepHO and LepFNR variants

**Fig 1:**
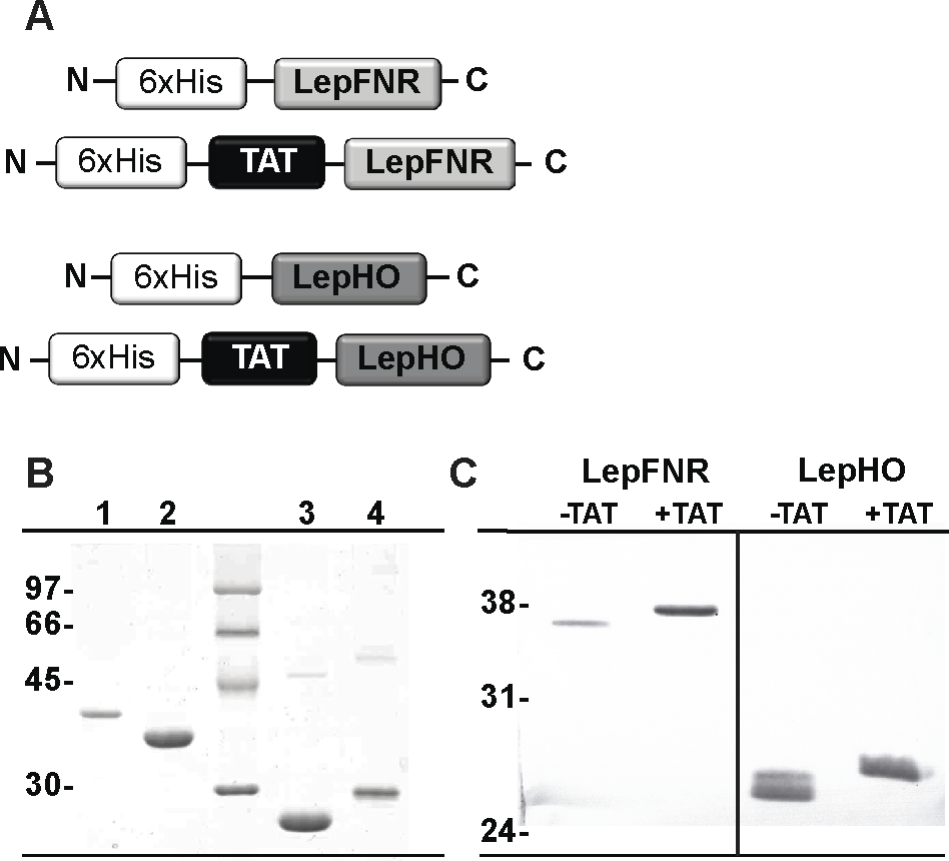
Protein design, expression and purification. (A) Schematic representation of wild type and TAT fusion forms of LepHO and LepFNR. (B) Purified fractions of each recombinant loaded into 12% SDS-PAGE and subsequently stained with Coomassie blue: lane 1, His-TAT-LepFNR (39.1 kDa); lane 2, His-LepFNR (37.6 kDa); lane 3, His-LepHO (28.4 kDa); Lane 4, His-TAT-LepHO (29.9 kDa). (C) Western blot analysis of purified products using primary antibodies anti-LepFNR and anti-LepHO raised in rabbits.

Expression system for the wild type and TAT fusion forms of LepHO and LepFNR were designed (Fig 1A). All proteins were obtained in soluble and pure forms with good yields, as judged by SDS-PAGE and Western blot analysis (Fig 1B).

**Fig 2:**
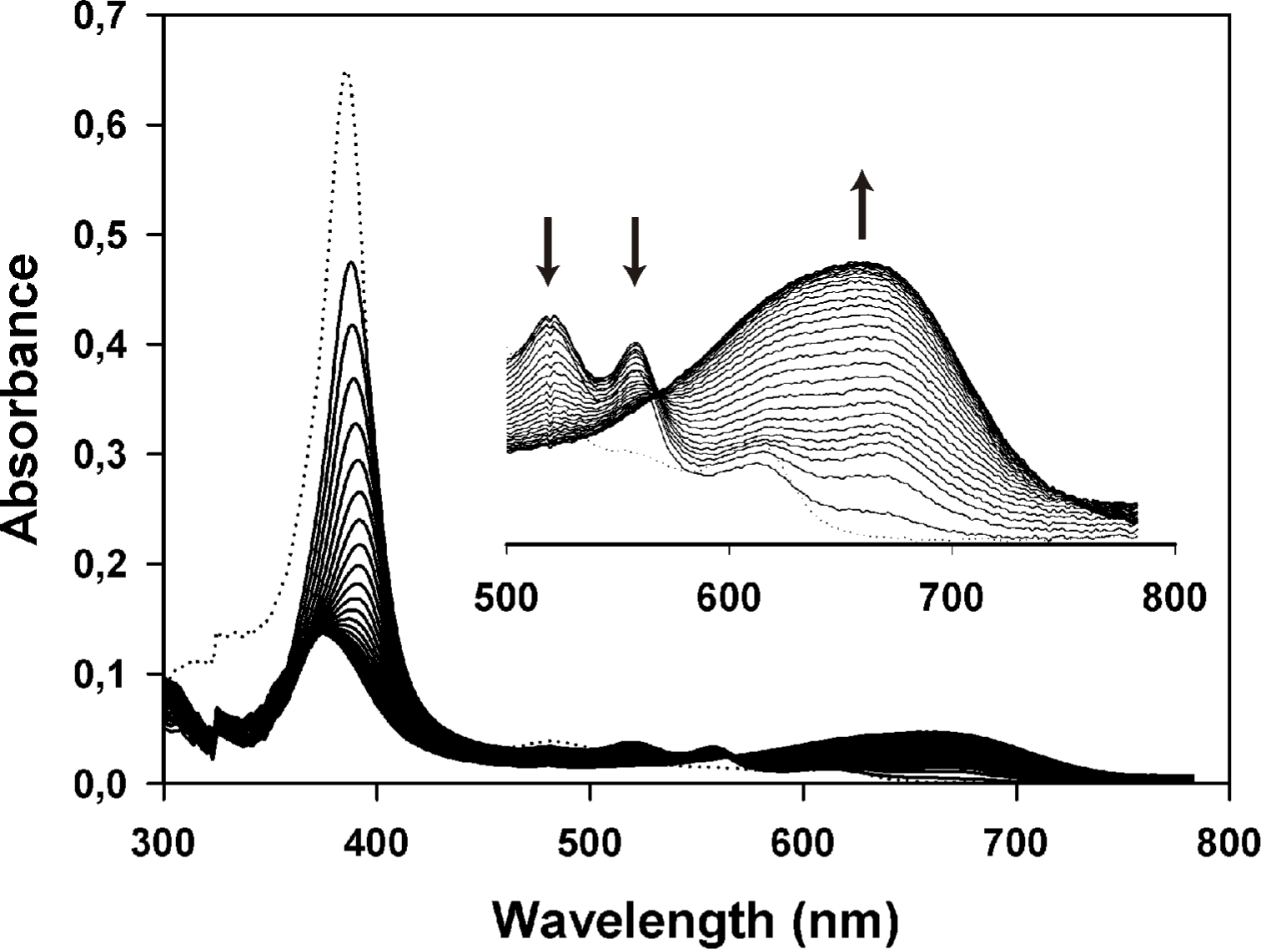
Heme turnover by TAT-LepHO in the presence of TAT-LepFNR. Time dependent absorption spectra of TAT-LepHO before (dotted line) and after the addition of 0.5 μM TAT-LepFNR in the presence of 300 μM NADPH and 0.1 mg/ml catalase recorded at 1 min intervals (solid lines). As the reaction proceeded, the TAT-LepHO Soret peak at 403 nm decreased, and a broad absorption band with maximum near 680 nm (biliverdin) appeared, indicating that TAT-LepHO channeled TAT-LepFNR electrons to cleave heme and produce biliverdin. The inset shows an enlargement of the region between 500 and 800 nm.

Heme turnover was spectrophotometrically determined to confirm that TAT extension does not alter electron transport between LepFNR/LepHO enzymes. Incubation of TAT-LepHO with TAT-LepFNR and NADPH resulted in spectral changes that are consistent with the formation of a ferrous dioxyheme complex [Heme(Fe^2+^)-O_2_] and the conversion of the latter to biliverdin. These can be seen in the time-dependent decay of the 538 and 575 nm peaks (ferrous dioxyheme complex) and growth of a broad band at 680 nm (biliverdin) (Fig 2).

### Characterization of TAT-LepHO and TAT-LepFNR transduction into SH-SY5Y cells

**Fig 3:**
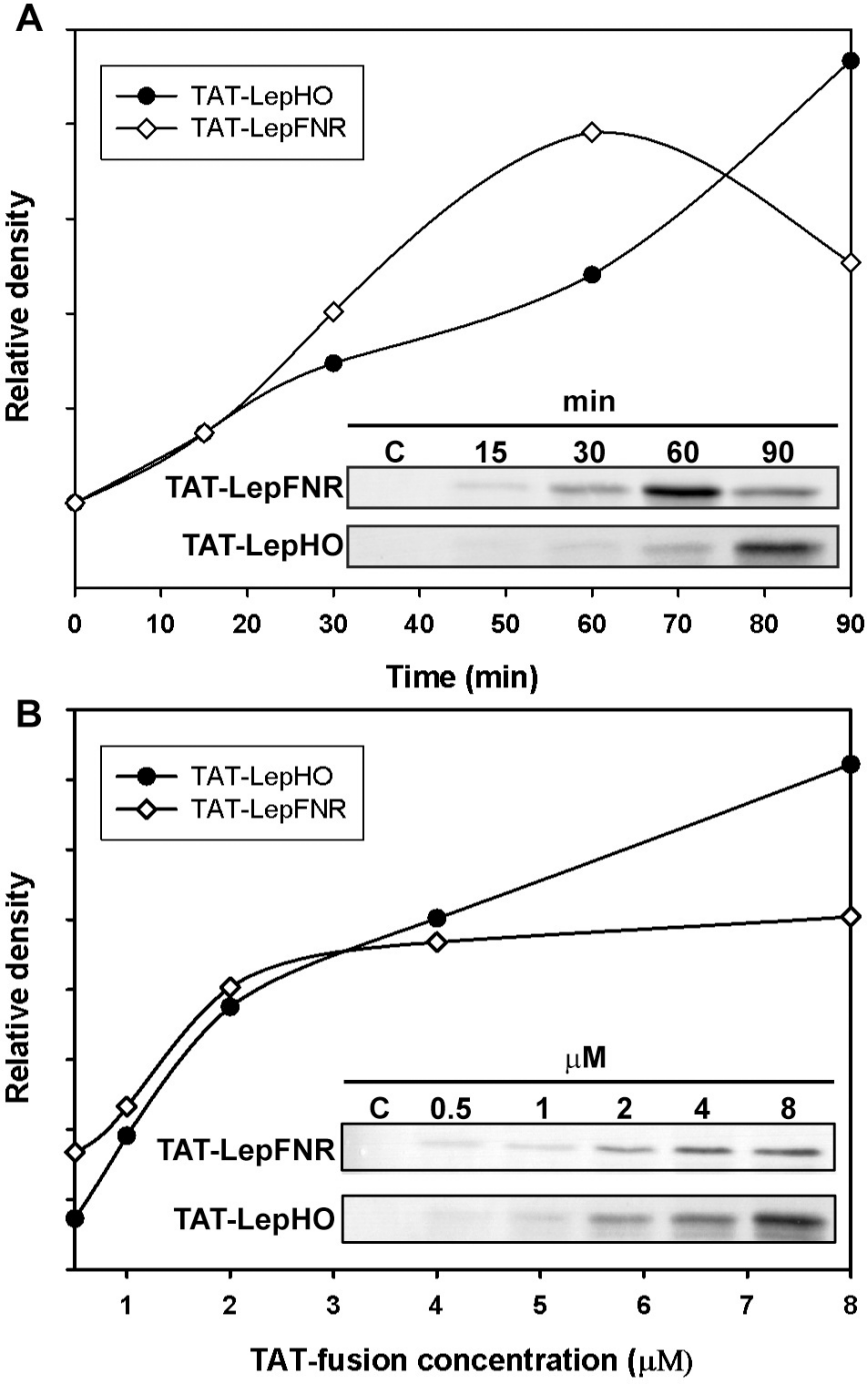
Transduction of TAT-LepFNR and TAT-LepHO into SH-SY5Y cells. (A) Transduction time dependence. SH-SY5Y cells were incubated with 1 μM TAT-LepFNR (white diamonds) or TAT-LepHO (black circles) in serum free medium. Cell extracts were prepared at different time intervals and examined by Western blot analysis. As a control, cells were incubated for 1 h with 1 μM Lep-FNR/Lep-HO. (B) Transduction concentration dependence. SH-SY5Y cells were incubated for 1 h with TAT-LepFNR (white diamonds) or TAT-LepHO (black circles) in serum free medium at stated concentration of each protein. Cell extracts were prepared and examined by Western blot analysis. As a negative control, cells were incubated for 1 h with 3 μM Lep-FNR/Lep-HO. Band density was calculated relative to a loading control of βIII-tubulin. Images are representative of 3 different experiments.

Purified TAT-LepHO or TAT-LepFNR (1 μM) were added to the culture medium. Samples were taken at various incubation times (15–90 min), and cells were exposed to trypsin to degrade the extracellular membrane-bound TAT-fused proteins. Then, the amount of internalized proteins of each sample was determined by Western blotting using anti-LepHO, LepFNR or βIII-Tubulin as a control. TAT-LepFNR was already detected inside the cells after 15 min of incubation. Uptake of this fusion protein was gradually increased up to 1 h of incubation. Nonetheless, band signal began to drop when incubation time exceeded 1 h indicating that the import occurs in a time limited window and that TAT-LepFNR has a limited stability inside the cell. Conversely, TAT-LepHO uptake started at 15 min and kept rising throughout the 90 min incubation (Fig 3A). We further analyzed TAT-fused protein transduction by testing different protein concentration in the incubation media within 1 h of treatment (Fig 3B). We observed that TAT-LepHO translocation increased with concentration in the range of 0.5–8 μM. Instead, when extracellular TAT-LepFNR concentration raised above 2 μM the amount of internalized protein did not change, suggesting a saturable process.

**Fig 4:**
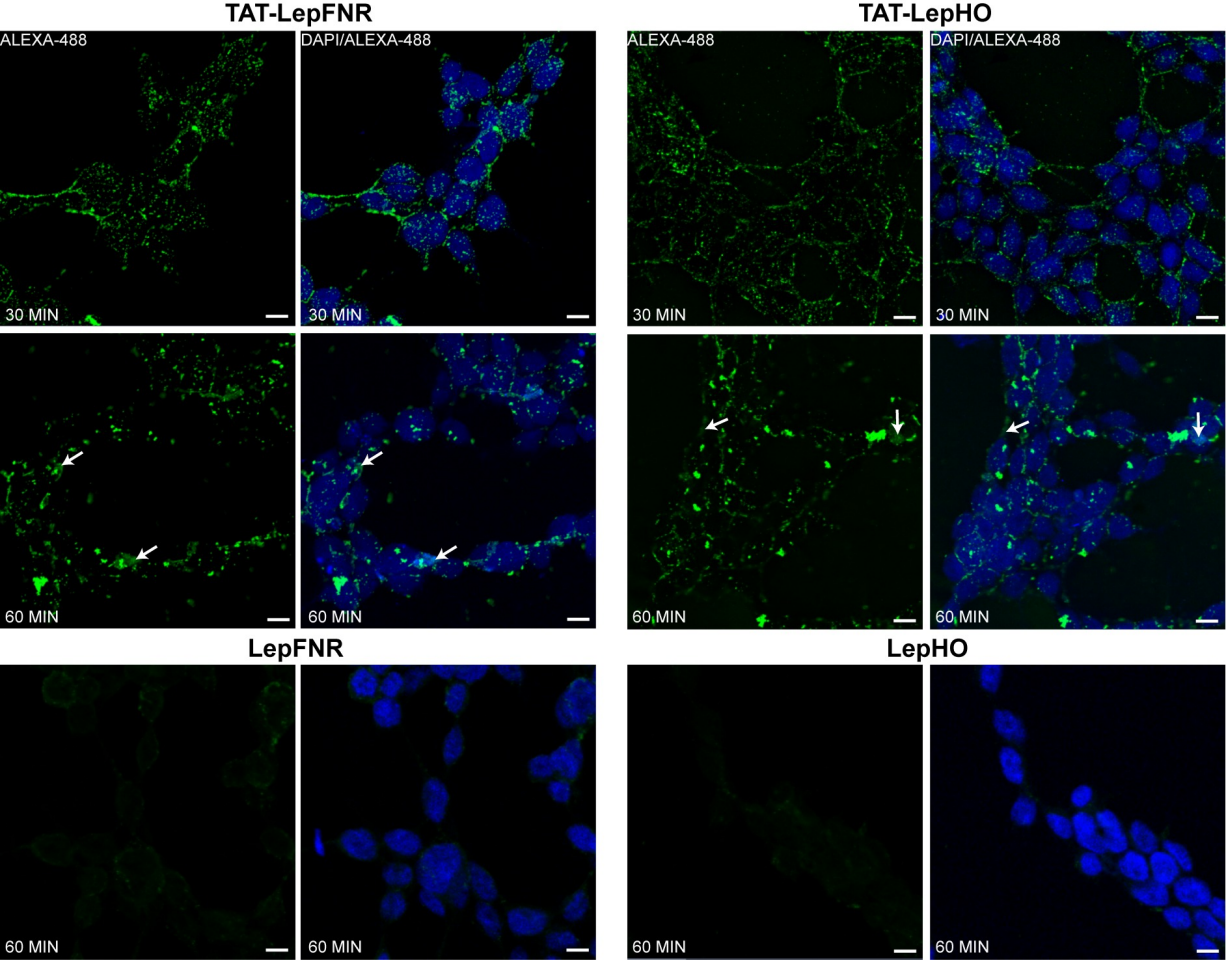
Immunofluorescence detection of transduced proteins. Cells were incubated with 3μM TAT-LepHO/TAT-LepFNR for 30–60 min using LepHO and LepFNR as negative controls, respectively. Fixed and permeabilized cells were then incubated with primary anti-LepHO/LepFNR antibodies. The coverslips were washed, incubated with the secondary Alexa-488 conjugated antibody, and mounted with ProLong with DAPI. White arrows show diffuse green fluorescent areas. Confocal images were obtained using a Zeiss LSM 880 confocal microscope with a 20X objective. Magnification: scan zoom x:3, y:3, scale bar 10μm. Zen image acquisition software (Carl Zeiss) was used to analyze images.

To confirm the TAT-linked protein penetration to cytoplasm and to track their destination, cells were incubated with TAT fusions of LepHO and LepFNR. Then, they were analyzed by immunofluorescence with anti-LepFNR and anti-LepHO antibodies followed by confocal laser scanning microscopy at different time points after the media was removed (Fig 4). We observed that after 30 min, both TAT-fused proteins were detected in cells cytoplasm in a very distinctive dotted pattern around the large nucleus of SH-SY5Y cells. At a longer time (60 min), however, some cells displayed areas of diffuse homogenous green pigmentation (white arrows); meanwhile, the population of small green dots had diminished and was replaced by larger ones, possibly as a result of the fusion of the smaller dots with each other. This might be attributed to a vesicle-like endosomal entrance in agreement with previous studies [9].

**Fig 5:**
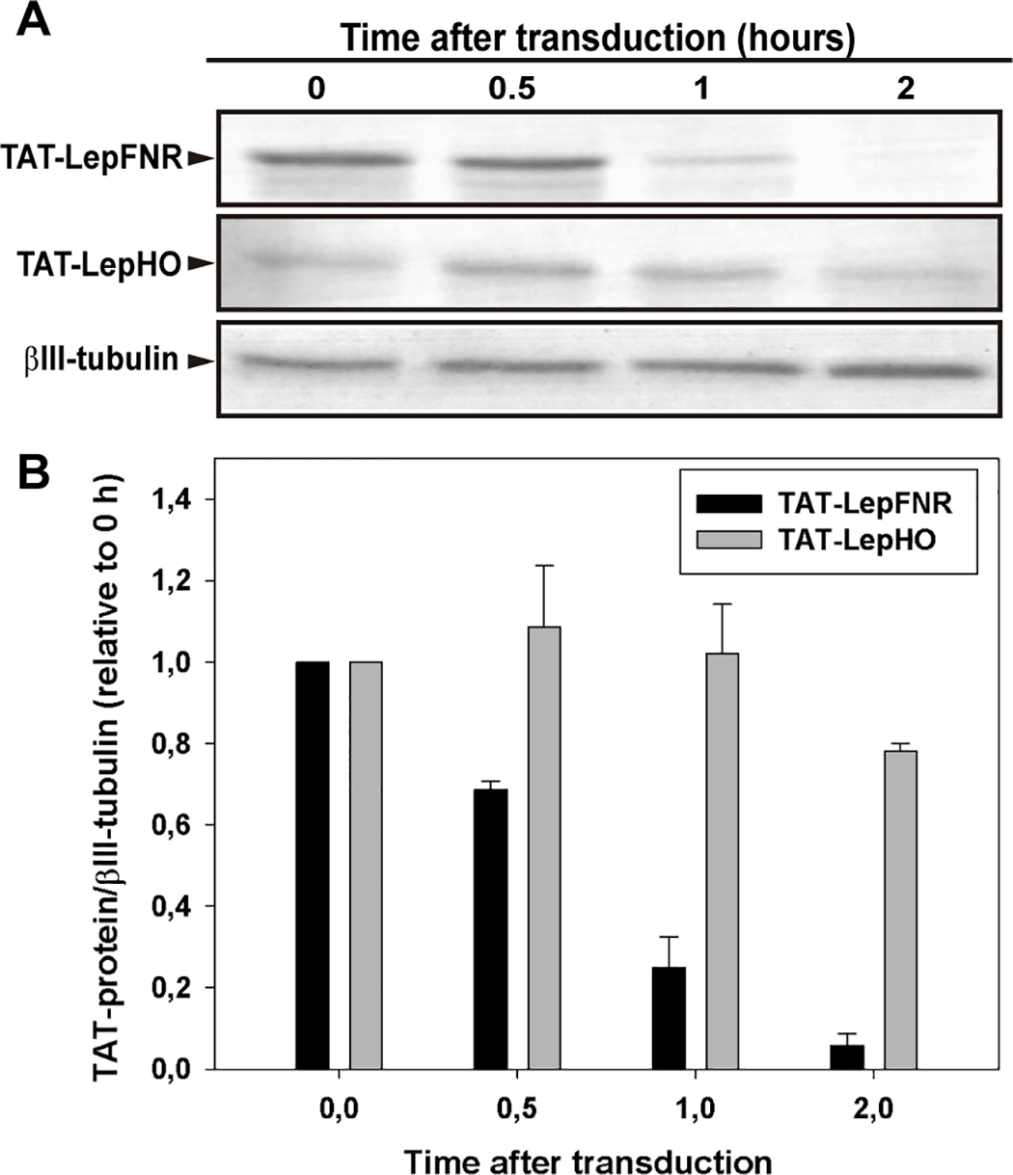
TAT-fusion protein stability inside the cell. (A) SH-SY5Y cells were incubated with 3 μM TAT-LepFNR or TAT-LepHO in a serum free medium for 1 h. The medium was removed and cells were washed with PBS buffer before a fresh medium was added. Cell extracts were prepared at different time intervals and examined by Western blot analysis. Representative blots of 3 different experiments are shown. (B) Densitometric analysis of the transduced proteins. Protein levels were normalized to that of βIII-tubulin.

The permanence of TAT-fused protein inside the cell was evaluated by 1 h cells incubation with TAT-protein (3 μM) in serum free media followed by medium removal, cell wash and further incubation in fresh DMEM-F12 for various time periods (Fig 5). We were able to detect TAT-LepFNR by western blot in total extracts obtained from cells collected 1 h post-incorporation, however, only traces of the imported protein were observed after 2 h. On the other hand, TAT-LepHO showed greater stability when compared to TAT-LepFNR.

**Fig 6:**
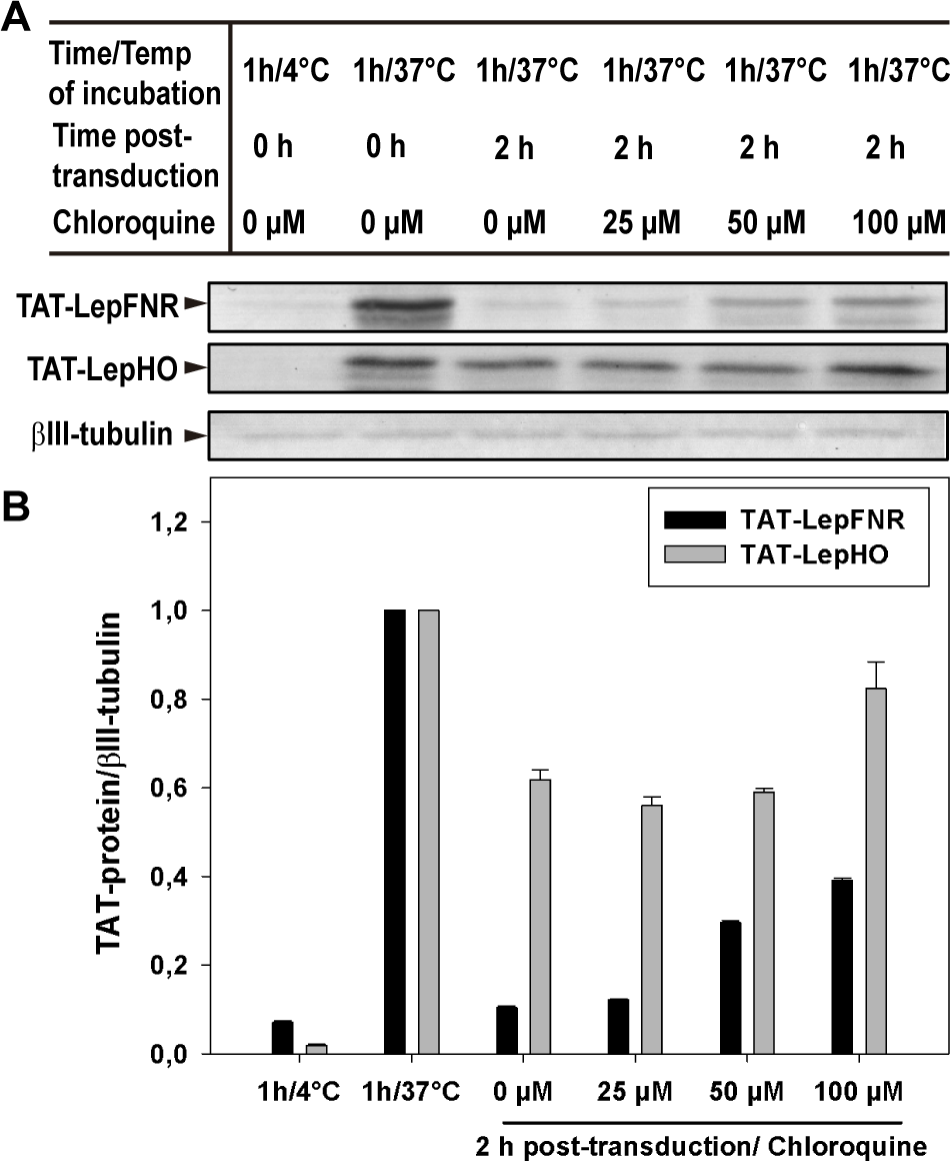
Transduction temperature dependence and chloroquine effects on the stability of the imported protein. (A) SH-SY5Y cells were incubated with 3 μM TAT-LepFNR or TAT-LepHO at 4°C and 37°C in a serum free medium. After 1 h of transduction Chloroquine was added to a fresh medium and incubation proceeded up to 2 h. Representative blots of 3 different experiments are shown. (B) Densitometric analysis of the transduced proteins. Protein levels were normalized to that of βIII-tubulin.

Many reports on TAT-PTD have pointed out that its entry mostly relies on endocytic pathways. An uptake decrease at low temperatures indicates that peptide internalization depends on an active membrane mechanism. Fig 6 shows that cells incubated at 4°C were not able to incorporate either TAT-LepHO or TAT-LepFNR, implicating that internalization of these macromolecules is a process that depends on plasma membrane fluidity. On the other hand, an increase of the protein transduction efficiency caused by chloroquine provides evidence of an endocytic route of entry. This endosomolytic reagent prolongs the fusion protein stability and promotes endosomal escape by delaying the endosome/lysosome fusion. Increasing concentrations of chloroquine markedly enhanced TAT-LepFNR permanence inside the cells. On the contrary, TAT-LepHO stability was virtually unchanged.

**Fig 7:**
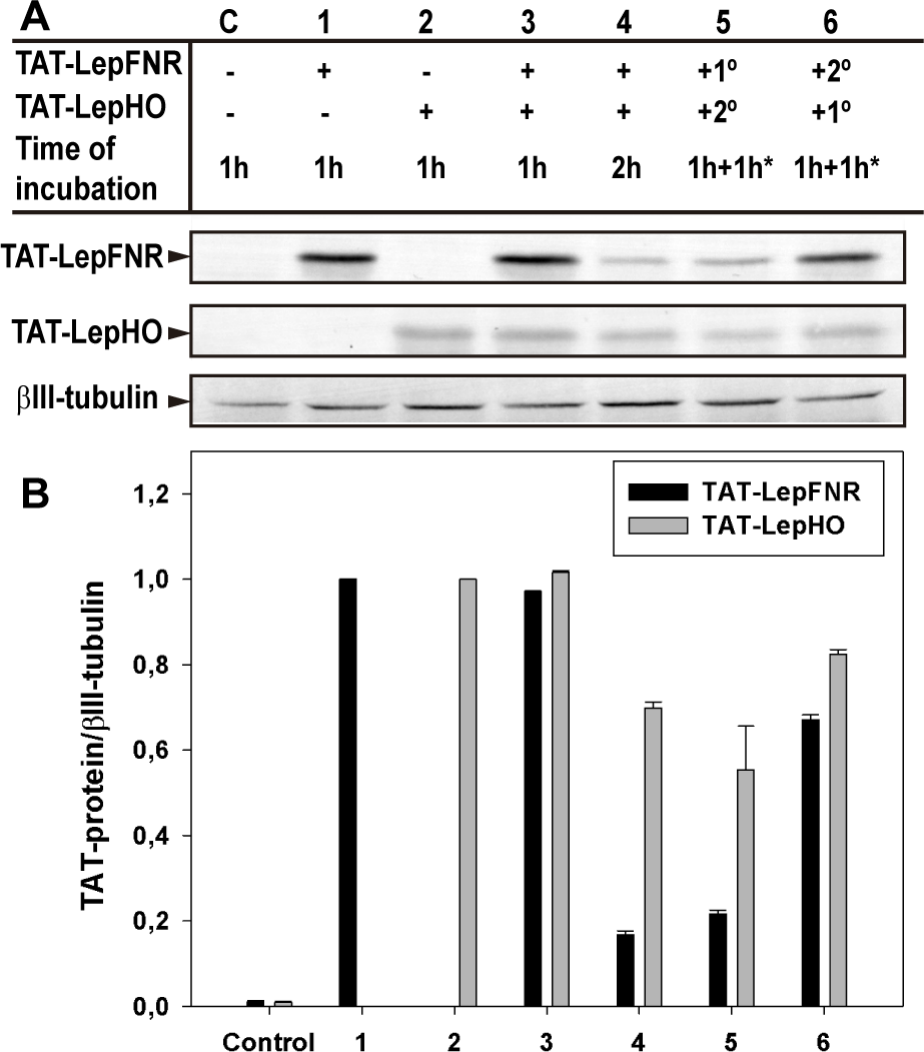
TAT-LepFNR and TAT-LepHO simultaneous transduction. (A) SH-SY5Y cells were incubated with each TAT-protein or both, as depicted, and cell extracts were prepared and examined by Western blot analysis. *The incubations were done sequentially in the indicated order. As a control (C), cells were incubated for 1 h with 1 μM Lep-FNR/Lep-HO (C). Representative blots of 3 different experiments are shown. (B) Densitometric analysis of the transduced proteins. Protein levels were normalized to that of βIII-tubulin.

SH-SY5Y cells were incubated with both TAT-fused proteins to investigate the transduction of each construct in the presence of the other. In this experiment, proteins were simultaneously added to the incubation medium, and cells were harvested after 1 or 2 h of treatment. Fig 7 shows that both TAT constructs were able to translocate the cell membrane at the same level they had done when they were individually added, implicating that they neither compete nor exhibit synergistic effects on their mechanisms of entry. When incubation extended up to 2 h, the level of TAT-LepFNR was drastically diminished in concordance with the previous experiments, indicating that TAT-LepHO has no effect on the internal stability of its co-transduced partner. Additionally, proteins were added sequentially to establish if the previously imported protein had any effect on the translocation of the other. Once again, it was demonstrated that they had little or no effect on the import process of each and that TAT-LepFNR permanence inside the cell is clearly shorter when compared to TAT-LepHO.

### Oxidative stress protection by simultaneous incorporation of TAT-LepFNR and TAT-LepHO

**Fig 8:**
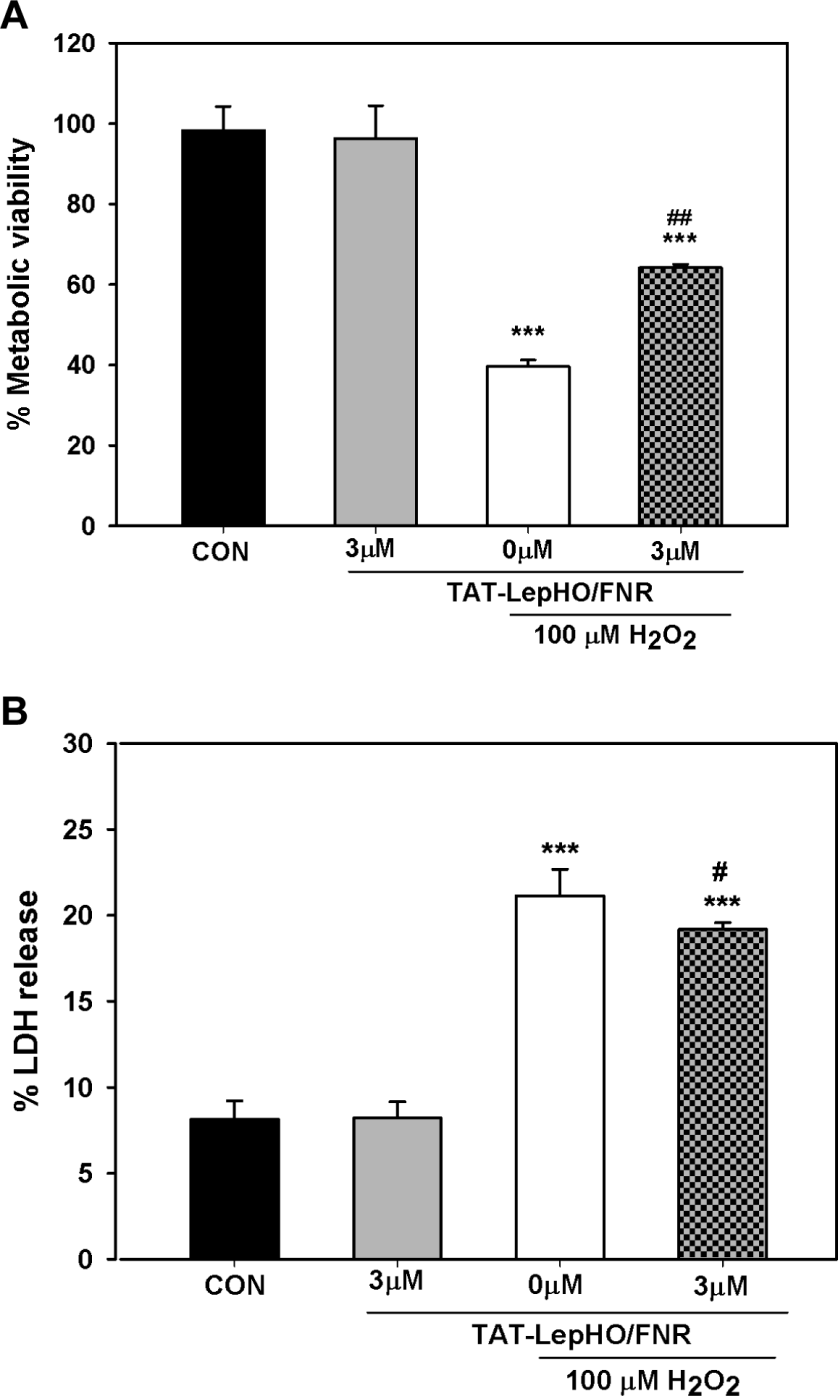
Rescue of cell viability after H_2_O_2_ exposure mediated by TAT-fused protein delivery. SH-SY5Y cells were exposed to 100 μM H_2_O_2_ and treated with TAT-LepHO and TAT-LepFNR, as described in Material and methods. Cell viability was measured as follows: (A) Metabolic viability. Culture media was replaced with 100 μL MTT solution and after 4 h, the formazan crystal produced was dissolved in DMSO and absorption at 540 nm was determined. Cell viability was expressed as percentage of control cells MTT reduction, n = 3. ***p<0.001 vs. CON (control) cells; ^##^p<0.01 vs. H_2_O_2_ alone treated cells. (B) LDH release. Aliquots of media and reagent were mixed in a 96-well plate and NADH absorbance decay was recorded at 340 nm. Data were expressed as a percentage of LDH released with respect to total LDH activity. The medium of cells treated with RIPA buffer represented Total LDH activity, n = 3. ***p<0.001 vs. CON (control) cells; ^#^p<0.05 vs. H_2_O_2_-alone treated cells.

The yellow tetrazolium MTT is reduced by metabolically active cells to a purple formazan. As previously stated, LepFNR and LepHO might work together in the prevention of oxidative stress injury and thus, this would reflect in the rescue of the metabolic status of cells. The metabolic viability of cells exposed to H_2_O_2_ (100 μM), as described in Material and methods, was 39.6 ± 1.6% of the control (p<0.001). However, when cells were additionally treated with TAT-LepHO/FNR (3 μM-18 h), metabolic viability significantly increased to 64.16 ± 0.8% (p<0.01) (Fig 8A). Assays of MTT reduction in cells treated with TAT-LepHO or TAT-LepFNR individually were performed (S1 Fig). TAT-LepFNR yield only *ca* 6% protection. Previous results have shown protection of Cos-7 cells against ROS by transient transformation with a plasmid that expressed pea ferredoxin NADP+ reductase [22]. The treatment of cells with TAT-LepHO displayed no significant differences with respect to the control.

To compare the efficiency of TAT-LepHO/FNR metabolic viability recovery with that produced by known natural antioxidants, vitamin C and E were analyzed. We found that vitamin C and E provoked a recovery of ~14% and ~18% respectively. Likewise, cells transduced with both fusion proteins displayed an ~22% protection (S2 Fig)

LDH release was evaluated as a marker of cytotoxicity induced by H_2_O_2_ exposure. Cells incubated in the presence of H_2_O_2_ (100 μM), as described in Material and methods, displayed an LDH activity increase of 21.1 ± 1.5% (p<0.001) when compared to 8.1 ± 1.0% released by the control group. When H_2_O_2_ treated cells were then incubated in medium containing TAT-LepHO/FNR (3 μM), LDH leakage decreased to 19.2 ± 0.4% (p<0.05), showing a statistically significant difference (Fig 8B).

These experiments suggest that LepHO and LepFNR TAT-mediated internalization resulted in a redox response enhancement that protected cells from oxidative stress damage.

**Fig 9:**
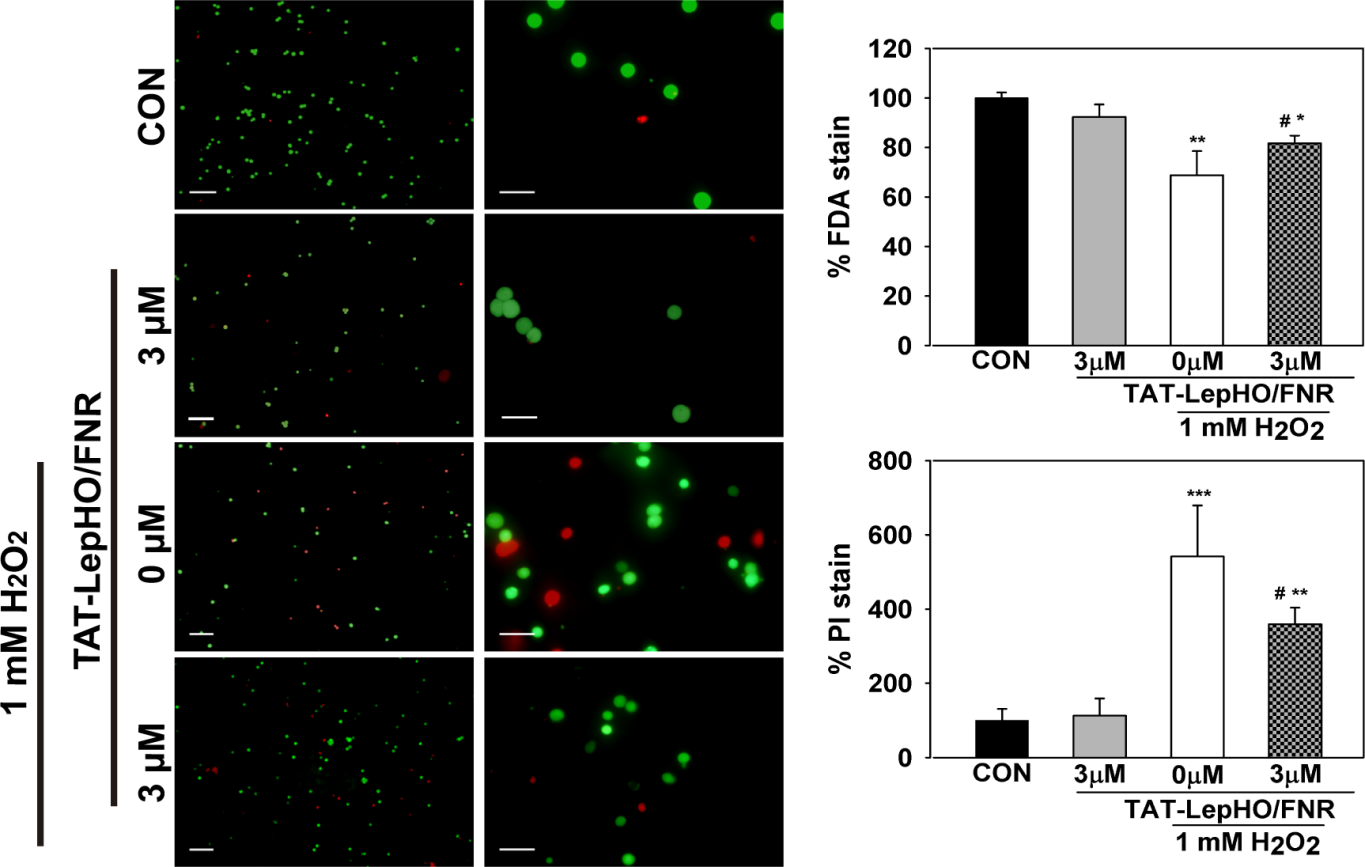
Vital staining FDA/PI showed increased H_2_O_2_ tolerance as a result of incubation with TAT-fused proteins. SH-SY5Y cells in suspension (2 x 10^6^) were simultaneously treated with 3 μM TAT-LepHO and TAT-LepFNR or left in serum free medium for 1 h and then exposed to 1 mM H_2_O_2_ for another hour. Cell pellets were washed with PBS buffer and resuspended in 500 μl of staining medium (serum free DMEM-F12, 2 μg/mL Fluorescein Diacetate, 1.2 μg/mL Propidium Iodide). n = 3, quantification was made using cell counting software (ImageJ) on 3 image fields of each independent experiment. *p<0.05 vs. CON (control) cells; **p<0.01 vs. CON (control) cells; ^#^p<0.05 vs. H_2_O_2_ alone-treated cells. Scale bar first column = 50 μm; scale bar second column = 20 μm.

Fluorescein Diacetate (FDA) is taken up by viable cells and enzymatically converted to the green fluorescent metabolite fluorescein in an esterase dependent reaction. In contrast, the nuclei staining dye Propidium Iodide (PI) only reaches the nucleus by passing dead cell membranes, and intercalates with the DNA double helix giving a red fluorescent signal. As shown in Fig 9, 68.8 ± 9.7% of the cells that had been exposed to growth media containing H_2_O_2_ (1 mM) were stained with FDA. Cells subjected to the same treatment displayed a 5.4-fold increase of PI stained nucleus (541.5 ± 137.4%) when compared to control cells (p<0.01). Then again, cells that were previously treated with TAT-LepHO/FNR (3 μM) for 1 h before H_2_O_2_ exposure, presented an elevated occurrence of green FDA stained cells (81.7 ± 3.1%) and decreased red PI stained nucleus (359.2 ± 44.2%) (p<0.01). The analyzed images indicated that transduction of redox active proteins might confer cells better odds to overcome an oxidative stress situation.

**Fig 10:**
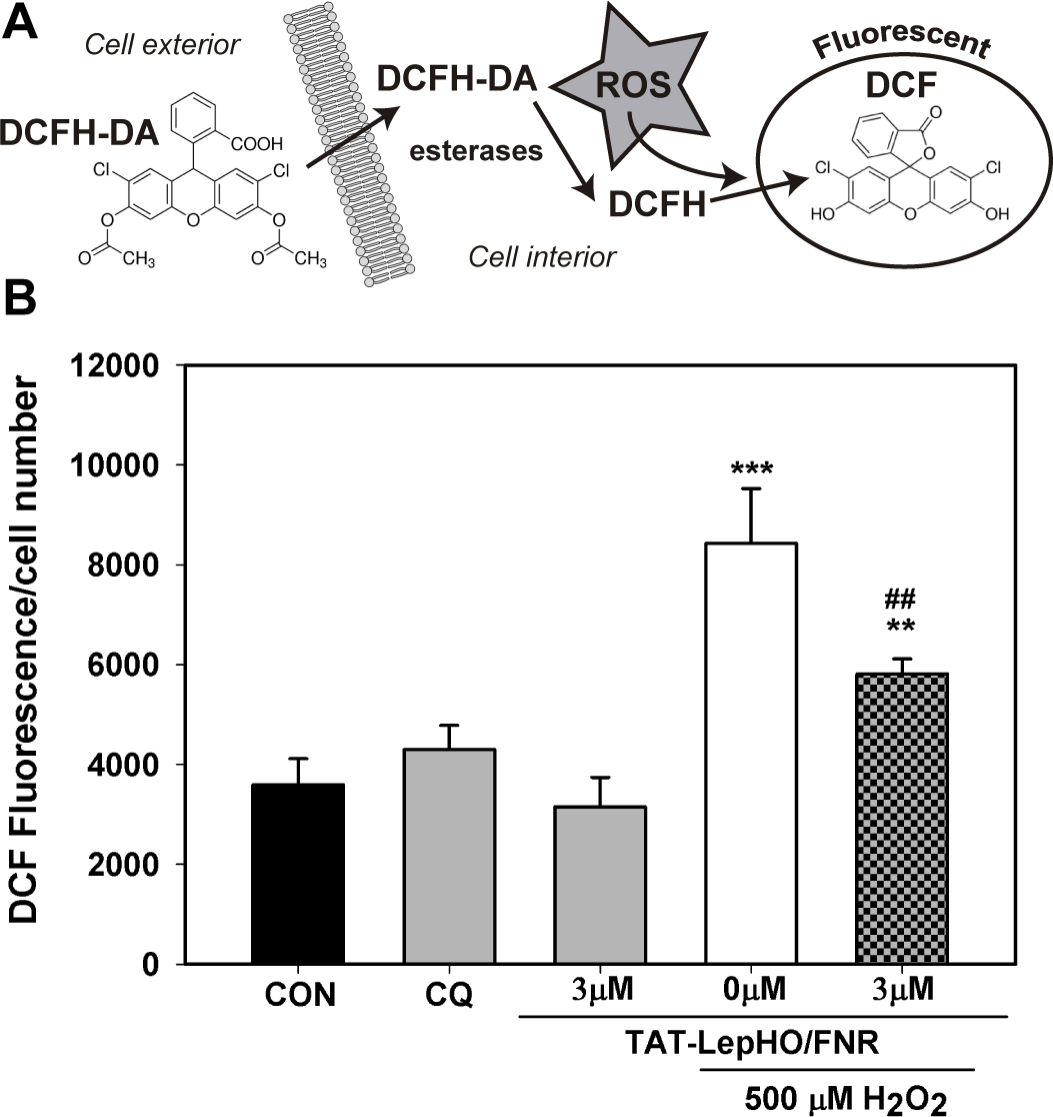
Intracellular ROS decrease mediated by TAT-fused protein delivery. (A) Strategy for reactive oxygen species quantification using DCFH-DA probe. (B) Cell monolayers were pre-treated for 2 h with 500 μM H_2_O_2_. Medium was discarded and cells were loaded with 80 μM DCFH-DA (30 min) and washed. Cells were either incubated in serum free medium alone or treated with 3 μM TAT fused proteins and 100 μM Chloroquine (1 h). CQ represents a control where DCFH-DA loaded cells were subsequently incubated with 100 μM chloroquine alone. After trypsinization, cells were resuspended in PBS buffer and transferred to a black 96 well plate to read fluorescence at 520 nm (excitation: 485 nm). n = 3. **p<0.01 vs. CON (control) cells; ***p<0.001 vs. CON (control) cells; ^##^p<0.01 vs. H_2_O_2_ alone treated cells.

The 2’,7’–dichlorofluorescein diacetate (DCFH-DA) probe is able to diffuse into the cell through the plasmatic membrane, being then deacetylated by cellular esterases to a nonfluorescent compound (DCFH), which is later oxidized to DCF (λ excitation = 498 nm; λ emission = 522 nm) by hydroxyl, peroxyl and other ROS activity within the cell (Fig 10A) [30]. This method has been shown to be reliable and efficient for the quantitative evaluation of oxidative stress in cells as well as in testing the efficacy of antioxidants [31].

Cells challenged with H_2_O_2_ (500 μM) for 2 h exhibited a 2.3-fold increment in DCF fluorescence (8428.1 ± 1095.8 RFU) relative to the control cells (3590.8 ± 521.2 RFU) (p<0.001). In contrast, cells exposed to H_2_O_2_, and subsequently treated with active redox fusions of TAT (3 μM), decreased DCF fluorescence readings to 5809.6 ± 304.0 RFU (p<0.01). These data indicated that simultaneous internalization of TAT-LepFNR and TAT-LepHO contributed to maintaining the redox balance, whereby diminishing ROS accumulation inside the cells (Fig 10).

## Discussion

The central role of cell membranes is to enable the exchange of information between the cell and its environment and to contain the essential components while acting as a barrier that protect cells against external factors. They also participate in numerous metabolic processes that are fundamental for cell survival. The membrane also imposes a serious obstacle for the import of various exogenous therapeutic molecules into the cell. Therefore, delivery of potential therapeutic agents constitutes a significant challenge to overcome. The finding of naturally occurring proteins that can enter cells, opened the door to a promising alternative in molecular delivery. Nowadays, CPPs or PTDs are widely used as research tools, and clinical trials are testing PTD-mediated delivery of macromolecular drugs in patients [32]. In the present study, we have demonstrated that two TAT-fused functional enzymes from *Leptospira interrogans* could be translocated simultaneously into neuronal cells and, therefore, enhance their tolerance to oxidative stress.

### Transduction of the fusion proteins under study mediated by TAT-PTD

We demonstrated that purified TAT-fused proteins were active as they reduced heme to biliverdin in the presence of NADPH (Fig 2). Moreover, they were readily internalized by cells after 15 min of incubation (Fig 3). TAT-LepFNR translocation as a function of its extracellular concentration suggested a cooperative and saturable process that might fit a glycosaminoglycan-mediated endocytosis mechanism. It is known that this import mechanism needs the recruitment of specific membrane components which help in the translocation process into the cell [10,33]. The turnover rate and availability of these membrane components could explain the behavior of TAT-LepFNR translocation. On the other hand, TAT-LepHO showed a linear relationship with time and did not reach an extracellular plateau concentration in our experimental conditions, indicating that the entry mechanism may vary depending on the cargo protein. Our transduction assays at 4°C showed that both constructions failed to enter cells at low temperature (Fig 6) suggesting that the uptake of the CPPs depends on a cellular process related to endocytosis rather than a direct translocation mechanism. Correspondingly, confocal images of cells incubated with the TAT-fused proteins showed a vesicle-like pattern around the nucleus (Fig 4). Furthermore, internal cell stability of TAT-LepFNR was remarkably lower than that observed for TAT-LepHO (Fig 5), and was affected by chloroquine treatment (Fig 6). Altogether, these results suggest that TAT-LepFNR could be internalized via an endocytic mechanism and that endosomal escape is a crucial step for its efficient delivery. Conversely, TAT-LepHO with a different chemical profile would exert its own particular interaction with membrane components explaining the discrepancies in the route of entry of TAT-fusions. Conjugating a cargo to a PTD modifies the overall charge, size, and hydropathy of the fusion, which will in turn define the character of the cell surface interaction, intracellular traffic, and final destination in the host cell as reported previously [13]. Additionally, we tested the simultaneous transduction of TAT-LepFNR and TAT-LepHO resulting in an effective uptake of both proteins (Fig 7). Our experiments clearly depicted that the nature of the cargo proteins influence the import mechanism mediated by PTDs as it has been reported before for CPP labeled with different dyes [34]. Therefore, for every molecule that needs to be translocated to the cytoplasm, CPP strategy should be carefully studied.

### Evaluation of TAT-fused proteins in oxidative stress protection

When neuronal cells harboring both TAT-fused proteins were exposed to H_2_O_2_ treatments, they showed a significantly reduced ROS accumulation and, therefore, a better tolerance to the oxidative stress, assayed as cell viability (Figs 8 and 9).

We observed that the protection induced by TAT-LepHO/FNR transduction measured by MTT reduction was higher than that determined by LDH release. It has been previously reported that different cytotoxicity assays might differ in their sensitivity to toxic stimulus [35]. ROS would damage cell components giving early signs of viability loss when testing MTT reduction. On the other hand, membrane leakage will occur in later stages as it involves a more profound damage to the cell. This could explain the fact that our treatment with H_2_O_2_ generates an important loss of MTT reduction capacity but a slight increase in LDH activity in culture media of treated cells.

It is known that NADPH participates on the oxidative protection through a complex system of metabolic response mechanisms [36]. An abnormal increase of NADPH level may be detrimental to cell survival. In yeast, it has been observed that NADPH accumulation is toxic for cell growth [37], a phenomenon probably common in all eukaryotes [38]. Cellular aggression by H_2_O_2_ generates important damage on enzymes involved in the NADPH metabolism and thus may alter its intracellular level. In bacteria, it has been proposed that an elevated NADPH concentration might have pro-oxidant effects by acting as an electron donor for the generation of hydroxyl radicals (OH^•^) via a Fe^2+^-dependent Fenton reaction [39]. This process may occur directly between the reduced pyridine nucleotide and the iron [40] or catalyzed by an enzyme that exhibits ferric reductase activity [41] also present in human cells [42]. Moreover, abnormal levels of NADPH could drive the generation of ROS by the NADPH oxidase. This enzyme complex is known to produce O_2_^-^ and/or H_2_O_2_ during the transfer of redox equivalents from cytosolic NADPH to molecular oxygen [43,44]. Thus, a possible explanation for the oxidative stress tolerance conferred by LepFNR might be related with the previously reported role of these flavoenzymes in reducing the NADPH intracellular concentration. FNR would prevent NADPH accumulation by mediating the electron transfer from NADPH to a variety of electron acceptors, as observed in bacteria [20,21]. Moreover, the transduced enzyme pair would have the capability to regulate the nucleotide level efficiently using the available cellular heme without the need for other electron acceptors.

Heme accumulation inside the cell produces diverse types of damages in enzymes and membranes which may drive to cell apoptosis. Then, by controlling its intracellular concentration, the damage can be mitigated [45]. The incorporation of the LepHO would produce an immediate decrease of the population free heme. LepHO is one of the heme oxygenases showing the highest affinity for heme, with a K_d_ for the prosthetic group of 17 nM [27]. Also, LepFNR possesses the ability to provide the redox equivalents necessary to complete the degradation of heme to biliverdin without the participation of any other auxiliary electron donors as ascorbate or trolox. Free heme can be converted by the LepFNR-LepHO pair in carbon monoxide and biliverdin with a concurrent release of Fe^2+^ which will induce the synthesis of ferritin for iron sequestration [46]. Moreover, converting heme to Fe^2+^ allows the cell to export the metal ion through the ferroportin [47], thus releasing the toxicity generated by heme or the free iron.

The products of LepHO degradation of heme may have other protective effects. Both biliverdin and bilirubin possess potent antioxidant properties [48,49]. Carbon monoxide, like nitric oxide, is a signal molecule and a gaseous modulator. It is involved in cytoprotective gene up-regulation mainly based on its ability to interact with the heme groups of hemoproteins [50]. It has also been reported a non-enzymatic signaling function for HO-1. This enzyme is involved in the regulation of gene expression and in the modulation of protein translation [51]. It has been shown that participates in a complex network of regulations, interacting with DNA repair proteins and transcription factors as Nrf2 and STAT3 [51]. Nrf2 immunoreactive fragment may have significant implications on oxidative stress gene regulation. Likewise, STAT3 regulates cell proliferation, migration, and invasion [51] thus may influence the recovery of cells under stress conditions. These effects may be adjusted by the cellular localization of HO. It has been demonstrated that endogenous HO can be found in the endoplasmic reticulum, mitochondria, caveolae and in the nucleus, as a full protein or as a inactive processed form [52], indicating that protective functions may not only be related to its enzymatic activity.

In conclusion, we have provided evidence of a novel approach to preventing oxidative stress damage to neuronal tissue through an evolutionarily conserved redox enzyme pair by making use of TAT protein transduction domain.

Our observations allow us to suggest a potential use of this system in the protection of tissues subjected to different stress conditions, such as ischemia, trauma, or surgery. Among the advantages of this approach, it should be mentioned that, unlike tissue transformation, this treatment could be applied transiently when needed, and then removed by the cell itself, leaving no trace in the treated tissue.

## Supporting information

S1 Fig Rescue of cell viability after H_2_O_2_ exposure mediated by TAT-LepHO, TAT-LepFNR or the pair TAT-LepHO/TAT-LepFNR. SH-SY5Y cells were exposed to 100 μM H_2_O_2_ and treated with TAT-LepHO, TAT-LepFNR or the pair TAT-LepHO/TAT-LepFNR as described in Material and methods. Metabolic viability was measured as follows: At the end of the 24 h incubation culture media was replaced with 100 μL MTT solution, the formazan crystal produced after 4 h was dissolved in DMSO and absorption at 540 nm was determined. Cell viability was expressed as percentage of control cells MTT reduction, n = 3. ***p<0.001 vs. all H_2_O_2_ treated groups; ^###^p<0.001 vs. H_2_O_2_ alone and individually delivered TAT-protein groups.
(TIF)

S2 Fig Rescue of cell viability after H_2_O_2_ exposure mediated by vitamin C or Vitamin E, and the pair TAT-LepHO/TAT-LepFNR. SH-SY5Y cells were exposed to 100 μM H_2_O_2_ and treated with the pair TAT-LepHO/TAT-LepFNR, vitamin C or E as described in Material and methods. Metabolic viability was measured as follows: At the end of the 24 h incubation culture media was replaced with 100 μL MTT solution, the formazan crystal produced after 4 h was dissolved in DMSO and absorption at 540 nm was determined. Cell viability was expressed as percentage of control cells MTT reduction, n = 3. ^###^p<0.001 vs. all H_2_O_2_ treated groups. ***p<0.001; **p<0.01; *p<0.05.
(TIF)

S1 Table Oligonucleotide sequences. (PDF)
