# Supplementary figures and images for: TAT-mediated transduction of bacterial redox proteins generates a cytoprotective effect on neuronal cells
Source: PLoS One. 2017 Sep 8;12(9):e0184617. doi: 10.1371/journal.pone.0184617 (PMC5591030; doi:10.1371/journal.pone.0184617)

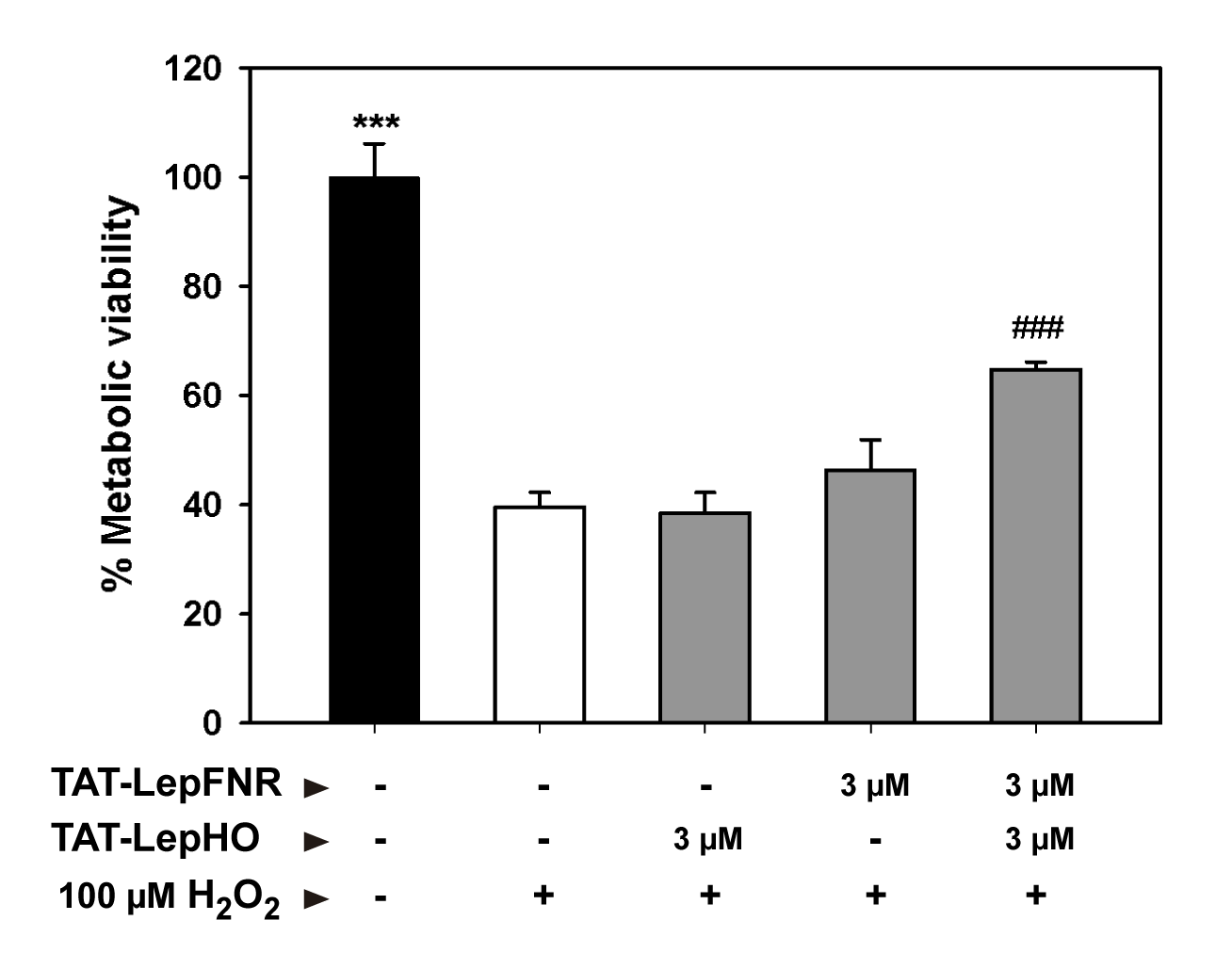

Supplement: S1 Fig — SH-SY5Y cells were exposed to 100 μM H2O2 and treated with TAT-LepHO, TAT-LepFNR or the pair TAT-LepHO/TAT-LepFNR as described in Material and methods. Metabolic viability was measured as follows: At the end of the 24 h incubation culture media was replaced with 100 μL MTT solution, the formazan crystal produced after 4 h was dissolved in DMSO and absorption at 540 nm was determined. Cell viability was expressed as percentage of control cells MTT reduction, n = 3. ***p<0.001 vs. all H2O2 treated groups; ###p<0.001 vs. H2O2 alone and individually delivered TAT-protein groups. (TIF) [file pone.0184617.s001.tif]

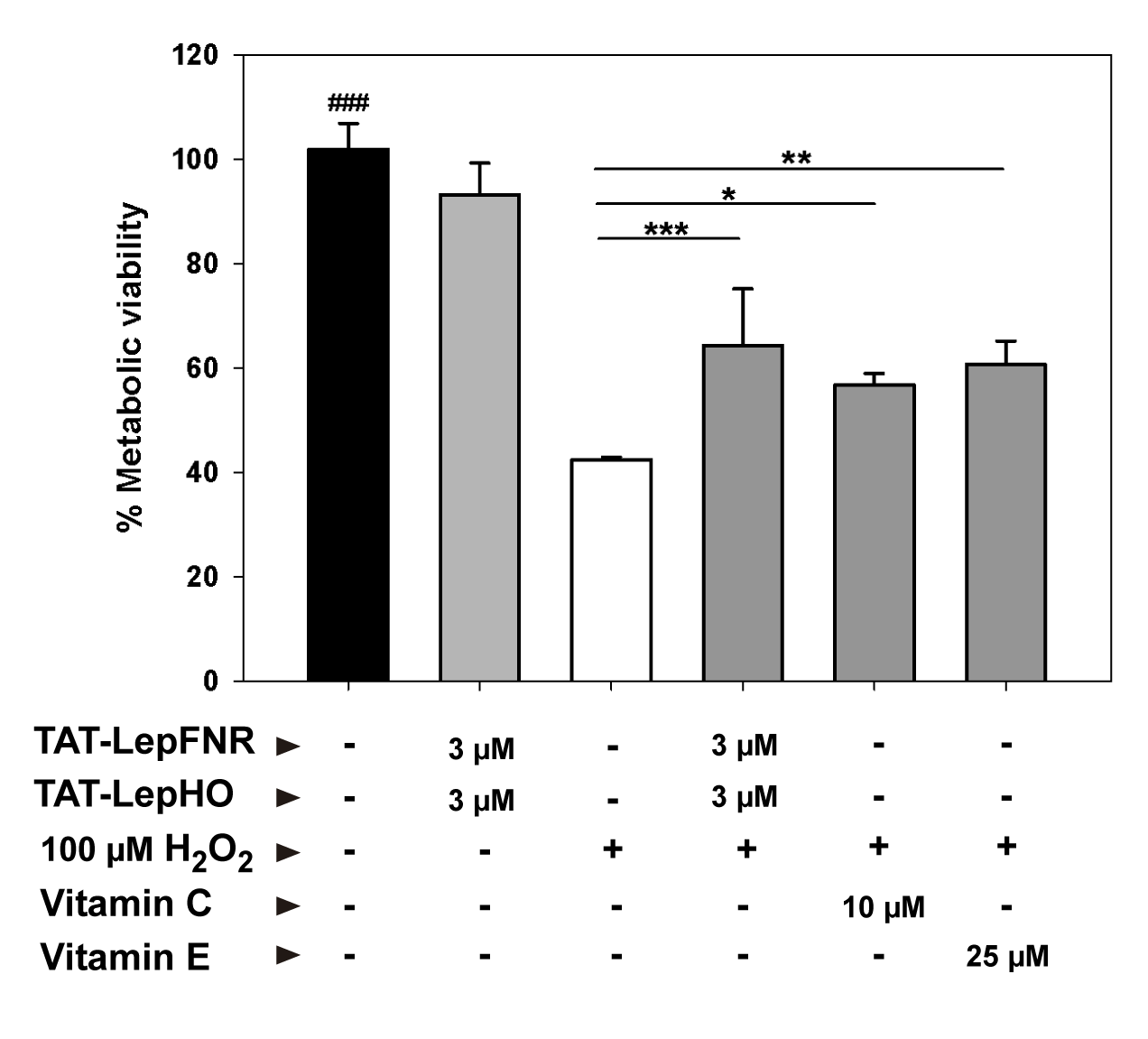

Supplement: S2 Fig — SH-SY5Y cells were exposed to 100 μM H2O2 and treated with the pair TAT-LepHO/TAT-LepFNR, vitamin C or E as described in Material and methods. Metabolic viability was measured as follows: At the end of the 24 h incubation culture media was replaced with 100 μL MTT solution, the formazan crystal produced after 4 h was dissolved in DMSO and absorption at 540 nm was determined. Cell viability was expressed as percentage of control cells MTT reduction, n = 3. ###p<0.001 vs. all H2O2 treated groups. ***p<0.001; **p<0.01; *p<0.05. (TIF) [file pone.0184617.s002.tif]
