# Supplementary material for: TAT-mediated transduction of bacterial redox proteins generates a cytoprotective effect on neuronal cells
Source: PLoS One. 2017 Sep 8;12(9):e0184617. doi: 10.1371/journal.pone.0184617 (PMC5591030; doi:10.1371/journal.pone.0184617)
Supplement: S1 Table — (PDF) [file pone.0184617.s003.pdf]

S1 Table. Oligonucleotide Sequences.

Oligonucleotides	Sequences (5'-3')
LepFNR-BamHI	GCGGGATCCATGCATTCGCTCATGAA
LepFNR-SacI	GCGGAGCTCTCAATATGTTTCCACAAATA
LepHO-BamHI	CGCGGATCCATGAGTTTAGCAACTATTTTACG
LepHO-HindIII	CCCAAGCTTTTAACCTTTTCCAAGAACGGAATC
TAT-NdeI	TATGTATGGCCGTAAGAAACGTCGACAGCGTCGCCGTG
TAT-BamHI	GATCCACGGCGACGCTGTCGACGTTTCTTACGGCCATACA
